# Dietary Plant Lectins Appear to Be Transported from the Gut to Gain Access to and Alter Dopaminergic Neurons of *Caenorhabditis elegans*, a Potential Etiology of Parkinson’s Disease

**DOI:** 10.3389/fnut.2016.00007

**Published:** 2016-03-07

**Authors:** Jolene Zheng, Mingming Wang, Wenqian Wei, Jeffrey N. Keller, Binita Adhikari, Jason F. King, Michael L. King, Nan Peng, Roger A. Laine

**Affiliations:** ^1^School of Nutrition and Food Sciences, Louisiana State University, Baton Rouge, LA, USA; ^2^Pennington Biomedical Research Center, Louisiana State University, Baton Rouge, LA, USA; ^3^Department of Veterinary Science, College of Agriculture, Louisiana State University, Baton Rouge, LA, USA; ^4^School of Life Sciences, Fudan University, Shanghai, China; ^5^Nicholls State University, Thibodaux, LA, USA; ^6^Louisiana Biomedical Research Network (LBRN) Summer Research Program (2010), Baton Rouge, LA, USA; ^7^Department of Biological Sciences, Louisiana State University and A&M College, Baton Rouge, LA, USA; ^8^Department of Chemistry, Louisiana State University and A&M College, Baton Rouge, LA, USA

**Keywords:** *Caenorhabditis elegans*, dopaminergic neurons, dopamine transporter, fluorescence, plant lectins

## Abstract

Lectins from dietary plants have been shown to enhance drug absorption in the gastrointestinal tract of rats, be transported trans-synaptically as shown by tracing of axonal and dendritic paths, and enhance gene delivery. Other carbohydrate-binding protein toxins are known to traverse the gut intact in dogs. Post-feeding rhodamine- or TRITC-tagged dietary lectins, the lectins were tracked from gut to dopaminergic neurons (DAergic-N) in transgenic *Caenorhabditis elegans* (*C. elegans*) [*egIs1*(*Pdat-1:GFP*)] where the mutant has the green fluorescent protein (GFP) gene fused to a dopamine transport protein gene labeling DAergic-N. The lectins were supplemented along with the food organism *Escherichia coli* (OP50). Among nine tested rhodamine/TRITC-tagged lectins, four, including *Phaseolus vulgaris* erythroagglutinin (PHA-E)*, Bandeiraea simplicifolia* (BS-I), *Dolichos biflorus* agglutinin (DBA), and *Arachis hypogaea* agglutinin (PNA), appeared to be transported from gut to the GFP-DAergic-N. *Griffonia Simplicifolia* and PHA-E, reduced the number of GFP-DAergic-N, suggesting a toxic activity. PHA-E, BS-I, *Pisum sativum* (PSA), and *Triticum vulgaris* agglutinin (Succinylated) reduced fluorescent intensity of GFP-DAergic-N. PHA-E, PSA, *Concanavalin A*, and *Triticum vulgaris* agglutinin decreased the size of GFP-DAergic-N, while BS-I increased neuron size. These observations suggest that dietary plant lectins are transported to and affect DAergic-N in *C. elegans*, which support Braak and Hawkes’ hypothesis, suggesting one alternate potential dietary etiology of Parkinson’s disease (PD). A recent Danish study showed that vagotomy resulted in 40% lower incidence of PD over 20 years. Differences in inherited sugar structures of gut and neuronal cell surfaces may make some individuals more susceptible in this conceptual disease etiology model.

## Introduction

Could dietary plant proteins, such as lectins, traverse the gut intact, with vesicular transfer to neurons and be transported intact along axons to affect dopaminergic neurons (DAergic-N) as one etiology of Parkinson’s disease (PD)? A recent Danish study showed that patients who had vagal nerves removed 20 years ago had a 40% lower incidence of PD (1). Some reports claim that vegetarians have higher rates of PD (2, 3). This study uses *C. elegans* as a model to investigate dietary lectin transport to DAergic-N.

Plant lectins were discovered over a century ago (4). Toxicity of some lectins was first recognized, independently, by Bruylants and Vennemann (5); Warden and Waddell (6) [described by Oppenheimer (7) and Dixson (8)]. Lectins’ hemagglutination properties were found by Stillmark (9), and a general recognition of antigenicity by lectins was revealed by Paul Ehrlich in 1890 (10) who won a Nobel Prize in Physiology or Medicine 1908 “*in recognition of their work on immunity*”^1^. Thereafter, lectins’ “immune recognition” was used for immunological research [see Textbook of Military Medicine (11)]. In 1919, Sumner crystallized (*Canavalia ensiformis*, Concanavalin A) (12). A half century later, investigators began to determine ABO-blood subtypes due to their sugar-binding properties, and the name “lectins” was formally coined (13, 14). Recent studies report that lectins play important roles in plant defense (15) and legume–rhizobial interactions (16).

Plants contain glycoprotein-lectins (“non-immune sugar-binding proteins”) in seeds, fruits, and nuts (2), and recognize and reversibly bind specific carbohydrates (17). They are involved in plant defense (15) and legume–rhizobia (16). Upon consumption by animals, they resist gut proteolytic enzymes, maintaining function under adverse gastrointestinal (GI) conditions (18, 19). They can penetrate the GI tract wall by endocytosis (20), probably by first binding a carbohydrate lectin receptor (21). Astonishingly, intact lectins can transfer trans-synaptically in an antegrade and/or retrograde fashion along nerve fibers (17, 22). Their medical importance is increasingly being recognized by being conjugated with drugs for better drug absorption from the GI tract (21, 23–25). Particularly relevant to the current studies, lectins have been utilized extensively for neuronal tracing studies [see review (22, 26)]. Ricin (*Ricinus communis*) as an extremely cytotoxic lectin has been studied extensively for its function in retrograde transport, *via* a galactose-binding β-chain-mediated endocytosis, following translocation of the enzymatically active and toxic A-chain, from the endosomes to the Golgi apparatus (27, 28). This property has been utilized for treatment of malignancies at low doses (29, 30). Lectins have also been conjugated with DNA for enhanced nervous system gene delivery (31). Most dietary plant lectins resist gut proteolytic enzymes and maintain function under usually adverse conditions for proteins (18, 19). Non-toxic lectins, such as tomato lectin and wheat germ agglutinin, are suggested to show growth factor activity in the GI tract (18). Bacteria or parasitic protozoa, through their own lectins, attach to carbohydrate receptors on epithelial cells to colonize the GI and genito-urinary tracts. Some lectins are synergistically toxic both locally and systemically to experimental animals (18). Kidney bean lectin (PHA), for example, damages intestinal epithelial cells, causes bacterial overgrowth, and induces nutritional disorders, effects which are preventable by inhibition with the specific sugars that have competitive binding capacities to lectins by sharing similar terminal structures (18, 32). Likewise, dietary saccharides or glycoconjugates, such as probiotic agents and milk oligosaccharides, may act as receptor analogs or decoys to selectively and competitively reduce lectin binding (18, 33, 34). Soybean lectin has shown potential anticarcinogenic effects (35).

Complex environmental factors play important roles for neurodevelopmental and neurodegenerative disorders, including PD (36, 37). Controversial reports suggest that a higher prevalence of PD occurs in vegetarians compared to omnivores (3, 38). In equine Parkinsonism, consuming yellow star thistles (*Centaurea solstitialis*) or Russian knapweed (*Acroptilon repens*) causes liquid necrosis in the *substantia nigra pars reticulata* and the *globus pallidus* by destroying DAergic-N, developing nigropallidal encephalomalacia (NPE), and creating histopathological features that resemble human idiopathic PD (39). These observations suggest transport of toxic substances from the horse gut to brain neurons. To date, however, in humans, epidemiology has not proven dietary lectins to have a significant impact on neuronal degenerative diseases. Signature pathologies of PD, e.g., Lewey’s bodies and aggregated alpha-synuclein (α-SYN) occur in neurons of the enteric nervous system of the GI wall, in addition to the neurons of the central nervous system (CNS) (40). α-SYN also aggregates in microglia and further leads to PD though the detailed mechanism remains unclear (41, 42), while astrocytes convert neurotoxin MPTP to its active metabolite MPP^+^ (43). The findings reported here support Braak and Hawkes’ hypothesis that the GI tract may be a potential site of neuronal invasion by an “unknown etiologic agent,” potentially responsible for causing some percentage of PD (40, 44–47). It is suggested herein that one possible etiologic agent could be dietary lectins.

*Caenorhabditis elegans* has a high conservation (>65%) of human disease-associated genes (48, 49). A total of eight DAergic-N in the hermaphrodite *C. elegans* (50–52) respond to signals from environmental mechano-sensory stimuli, e.g., exhausted food supply, which have offered molecular, genetic, and behavioral tools to aid human disease studies (53–57). *C. elegans* modulates locomotion behavior by using dopamine and serotonin to mediate motor circuits in chemical synapses, gap junctions, and neuromuscular junctions (58–60). Intestinal muscle cells are innervated by pharyngeal motor neurons and bioaminergic neurons *via* the preanal ganglia. Structures, sensory-motor synapses, gap junction contacts, and activities all resemble those in the mammalian GI tract (61, 62).

The features of the green fluorescent protein (GFP)-dopamine transporter (DAT) fusion protein *C. elegans* [*egIs1*(*Pdat-1:GFP*)] were evaluated by the numbers, fluorescent intensity, and sizes of GFP-DAergic-N in this study. Meanwhile, TRITC-labeled lectins were also followed post-feeding to establish the ability of lectins to bind or penetrate the GI wall or nerve cells. The question was whether dietary plant lectins can be transported to, and impair or alter apparently DAergic-N. Differences in inherited sugar structures of gut and neuronal cell surface may make some individuals more susceptible in this conceptual disease model.

## Materials and Methods

*Caenorhabditis elegans* (*egIs1*[*Pdat-1:GFP*]) that express GFP in the eight DAergic-N (63, 64) and the standard food *Escherichia coli* (*E. coli*) were obtained from *C. elegans* Genetics Center (CGC, MN). The *C. elegans* model does not require regulation of the Institutional Animal Care and Use Committee (IACUC).

### *C. elegans* Culture

*Caenorhabditis elegans* were egg-synchronized, fed *ad libitum* with LB broth (200 μl/agar plate or 250 μl/well) containing OP50 5 × 10^8^–5 × 10^11^ cfu/ml (65), grown in nematode growth media (NGM) agar plates (∅35 mm, 3 ml) and transported to new plates every other day (*n* = 20). Or, seeded in 96-well plate (*n* = 10–15/well) grown in liquid culture supplemented with fluorodeoxyuridine (FUDR, 0.6 mM/30 μl) (66). The plate was tape sealed, bagged, and covered with aluminum foil, and kept in a 20°C low temperature incubator (Revco Tech., Nashville, NC, USA) throughout the experiments.

All treatments were applied at day 3 after hatching. Four dose–responses of nine lectins were obtained for each culture condition in a dark room. Control animals were fed with OP50. Experimental groups were fed TRITC/rhodamine-conjugated lectins. The lectins were incorporated into feeding medium with OP50. Two hundred microliter treatments were added into each fresh agar dish prior to the transfer or 5 μl/well/week to liquid culture. Each group of nematodes was collected and fixed by 15–20 days for the agar dish culture as previously described (67), after the first week for the liquid culture.

### Culture of *Escherichia coli* (OP50)

OP50 (10 μl) and streptomycin (10 μl/ml) were mixed with LB Broth (100 ml, see below) for 16 h at 37°C in an incubator and stored at 4°C for up to 3 months.

### Select Lectins

Commercially available plant lectins conjugated to TRITC or rhodamine were from EY labs (San Mateo, CA, USA), Vector Labs (Burlingame, CA, USA), or Sigma-Aldrich (St. Louis, MO, USA). Doses of lectins (millimolar) used were comparable to those used in published work in neuronal tracing (17, 22, 26).

### Average Probability of Survival Assay

All average probability of survival (APS) assays was conducted in liquid culture (96-well plate). The animals were synchronized and seeded into each well of a plate (*n* = 10–15) and OP50 was added to each well. Thirty microliters (0.6 mM) of FUDR were added to each well to sterilize the animals. Four different treatments of lectins (50 μl/treatment, *n* = 6 row) were added. The plate was then covered with aluminum foil. The whole procedure was performed in a dark room to prevent bleaching of fluorophores. The survival animals were counted every other day until all were dead.

### Fluorescent Microscopy

The GFP-DAergic-N were identified by FTIC filter (480Ex/520Em) and the number of GFP-DAergic-N counted. Fluorescent intensity of GFP-DAergic-N and their average sizes (square micrometer) were determined by NIS-Elements Advanced Research (version 3.22.11) and compared among the following groups: control and lectins. Fluorescent intensity of rhodamine-lectins was determined by a TRITC filter (580Ex/620Em) to assess co-localization. The magnitude of the effect(s) of the lectin on the DAergic-N, the number, fluorescent intensity (arbitrary unit), and sizes (square micrometer) of GFP-DAergic-N were determined and compared among each group. Co-localization was initially identified with an inverted microscopy (Nikon, Eclipse Ti–S, Japan) and then confirmed at a *Z*-axle with laser scanning microscopy (Leica, TCS SP5, Germany).

### Solutions and Chemicals

Standard *NGM agar plates* (g): NaCl 3.0 g, Bacto-agar (Becton, MD, USA) 20 g, Bacto-peptone 2.5 g (Becton, MN, USA), Cholesterol solution 0.1% (0.005/ml 95% ethanol), and dH_2_O 975ml were mixed. Additions to the autoclaved solution (M): CaCl_2_ 1.0 1 ml, MgSO_4_ 1.0 1 ml, KPO_4_ pH6 1.0 25 ml. *LB Broth*: 25.0 g, dH_2_O 1 l (autoclave). *S-basal solution* (M): NaCl 0.1, KPO4 pH6 0.05, Cholesterol 0.1%, was autoclaved. *PBS* (millimolar): 115 NaCl, 75 Na_2_HPO_4_•7H_2_O, and 7.5 KH_2_PO_4_, pH 7.4.

### Statistical Analyses

Analyses were carried out using SAS/STAT^®^ software, Version 9.4 of the SAS System for Windows (Cary, NC, USA). All results were expressed as mean ± SEM. Survival curves were displayed by binomial probabilities obtained from logistic regression models as surrogates for survival probabilities and mean lifespan was estimated via Kaplan–Meier (log rank). ANOVA models were used to analyze fluorescence intensity data. For each group, 20 animals were analyzed for agar culture and 10–15 animals were analyzed for liquid culture. Statistical significance was defined as *P* < 0.05.

## Results

Diets supplemented with varying concentrations of rhodamine-labeled lectins *Phaseolus vulgaris* erythroagglutinin (PHA-E), *Bandeiraea simplicifolia* (BS-I), or *Dolichos biflorus* agglutinin (DBA) in agar dish, or TRITC-conjugated *Arachis hypogaea* agglutinin (PNA) in liquid culture were fed to *C. elegans*, and subsequently detected by fluorescence microscopy associated with GFP-DAergic-N (Table 1). The only explanation for this observation is that rhodamine- or TRITC-labeled lectins traveled in some manner from the gut to the neurons. We observed that some lectins had the following effects: (a) reducing the number of DAergic-N, (b) decreasing fluorescent intensity of GFP-expressing neurons (less GFP-DAT), or (c) altering neuron size. GSL-I, Con A, *Pisum sativum* (PSA), WGA, or S-WGA were not detected as transported to neurons, but nevertheless, some had significant effects on the neuron measurements, possibly indicating that undetectable amounts of these lectins caused the effects, or that some unexplained secondary effect of the lectins caused pathological effects.

**Table 1:** **Lectins detected in the neurons by co-localization**.

Lectins	Dose (mM)	GFP #		GFP intensity		GFP size	
*Phaseolus vulgaris* (PHA-E)-rhodamine	2.0 × 10^−4^	↓	*P* > 0.05	↓	*P* < 0.01	↓	*P* > 0.05
	6.0 × 10^−4^		*P* > 0.05		*P* < 0.01		*P* > 0.05
	2.0 × 10^−3^		*P* < 0.01		*P* < 0.01		*P* < 0.01

*Bandeiraea simplicifolia* (BS-I)-TRITC	5.3 × 10^−4^	↓	*P* > 0.05	↓	*P* < 0.01	↑	*P* < 0.01
	1.8 × 10^−3^		*P* > 0.05		*P* < 0.01		*P* < 0.01

*Dolichos biflorus* (DBA)-rhodamine	1.0 × 10^−3^	↔	*P* > 0.05	↔	*P* > 0.05	↔	*P* > 0.05
	3.3 × 10^−3^						

*Arachis hypogaea* agglutinin (PNA)-TRITC	1.8 × 10^−5^	↓	*P* > 0.05	↓	*P* < 0.01	↓	*P* > 0.05
	5.4 × 10^−5^		*P* > 0.05		*P* > 0.05		*P* > 0.05
	1.8 × 10^−4^		*P* < 0.01		*P* < 0.01		*P* < 0.01

*↓ Decreasing trend*.

*↑ Increasing trend*.

*↔ No significant alternation*.

### Lectins Co-Localized with the GFP-DAergic Neurons

**Figure 1:**
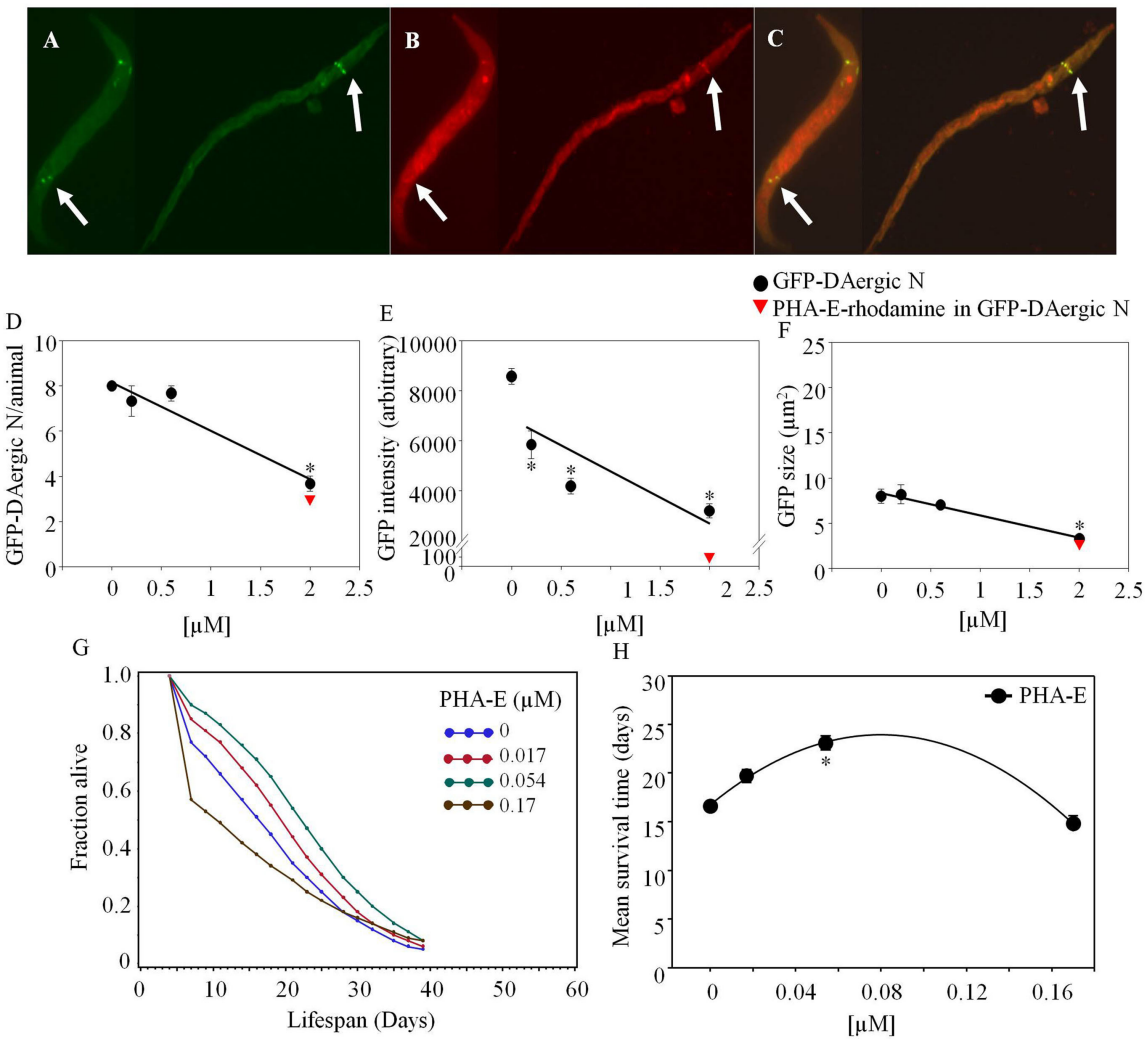
***Phaseolus vulgaris* (PHA-E)-rhodamine co-localized and diminished GFP-DAergic neurons in *C. elegans* post feeding**. **(A)** GFP-DAergic neurons. **(B)** PHA-E-rhodamine in several GFP-DAergic neurons. **(C)** Merged **(A,B)** showing co-localization of the PHA-E-rhodamine with GFP-DAergic neurons. **(D)** Dose-dependent reduction of the number of GFP-DAergic neurons per animal: 4.4 ± 0.3 (2 × 10^−3^ mM, *n* = 3, *P* < 0.01). **(E)** Dose-dependent reduction of fluorescence intensity per GFP-DAergic neurons: 5,837 ± 556 (2 × 10^−4^–2 × 10^−3^mM, *n* = 3, *P* < 0.01). **(F)** Size reduction of GFP-DAergic neurons: 3.3 ± 0.4 μm^2^ (2 × 10^−3^ mM, *n* = 3, *P* < 0.01). **(G)** The APS was increased dose-dependently. **(H)** Mean survival time of each group. *indicates statistical significance.

*Phaseolus vulgaris* erythroagglutinin-rhodamine co-localized with GFP-DAergic-N within 2 weeks after feeding (Figure 1). The number of GFP-DAergic-N was reduced in a dose-dependent manner (*P* < 0.01, Figure 1D). PHA-E-rhodamine co-localized to a subgroup of GFP-DAergic-N (Figure 1B). The fluorescence intensity of GFP-DAergic-N was decreased dose-dependently (*P* < 0.01, Figure 1E), suggesting a diminution of the GFP-DAT. The average size of GFP-DAergic-N was also reduced at the highest dose (*P* < 0.01, Figure 1F). PHA-E-rhodamine fluorescence image size was inversely proportional to the number, average intensity, and average size of the GFP-DAergic-N. The APS was increased dose-dependently (Figure 1G). The mean lifespan was increased at a medial dose (5.4 × 10^−5^ mM) from 17 to 23 days (39%, *P* < 0.05, Figure 1H).

**Figure 2:**
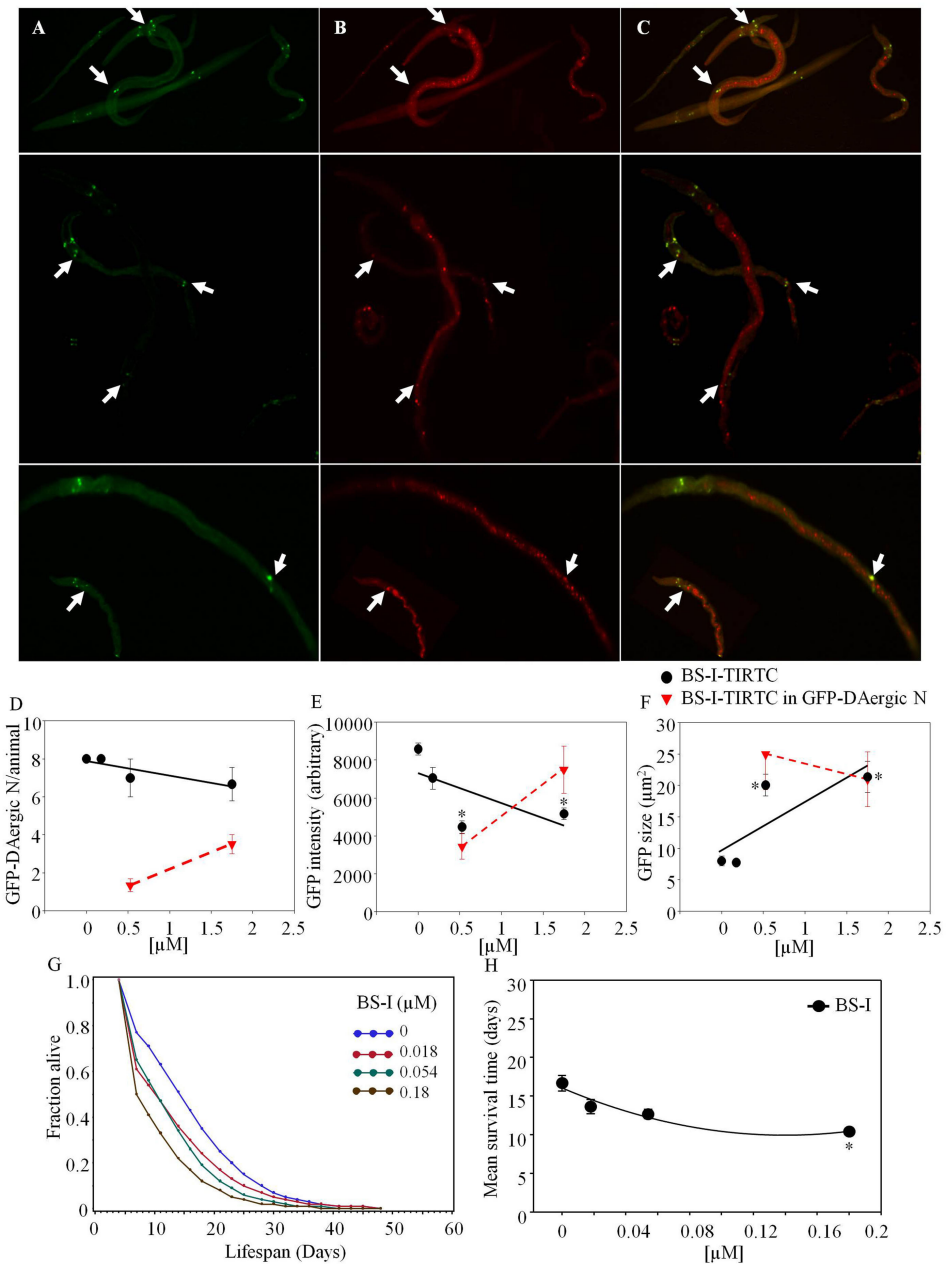
***Bandeiraea simplicifolia* (BS-I)-rhodamine co-localized with GFP-DAergic neurons: (A) GFP-DAergic neurons**. **(B)** BS-I-rhodamine in GFP-DAergic neurons (5.26 × 10^−4^ or 1.75 × 10^−3^ mM). **(C)** Merged **(A,B)** showing co-localization of the BS-I-rhodamine with GFP-DAergic neurons. **(D)** Dose-dependent reduction of GFP-DAergic neurons per animal: 7.0 ± 1.0 (1.75 × 10^−4^ mM) or 6.7 (1.75 × 10^−3^ mM) (*n* = 3, *P* > 0.05). **(E)** Reduction in fluorescence intensity per GFP-DAergic neuron (up to 60%): 4,468 ± 332 or 5,166 ± 300 (5.3 × 10^−4^ or 1.75 × 10^−3^mM, *n* = 3, *P* < 0.01). **(F)** The size of GFP-DAergic neurons was elevated dose-dependently from 8.0 ± 0.8 to 20.1 ± 1.7 μm^2^ (5.26 × 10^−4^) or 21.4 ± 2.5 μm^2^ (1.57 × 10^−4^) (*n* = 3, *P* < 0.01). **(G)** The APS was dose-dependently decreased at all doses. **(H)** Mean survival time of each group. *indicates statistical significance.

*Bandeiraea simplicifolia*–TRITC (Sigma-Aldrich) co-localized with GFP-DAergic-N (Figure 2). The number of the GFP-DAergic-N was reduced in a dose-dependent trend (*P* > 0.05, Figure 2D). The fluorescence intensity of GFP-DAT protein in DAergic-N was dose-dependently reduced (*P* < 0.01, Figure 2E). The size of GFP-DAergic-N was elevated (*P* < 0.01, Figure 2F). The APS was dose-dependently decreased at all doses (Figure 2G). Mean lifespan was decreased at the highest dose (1.8 × 10^−4^ mM) from 17 to 10 days (−37%, *P* < 0.05, Figure 2H).

**Figure 3:**
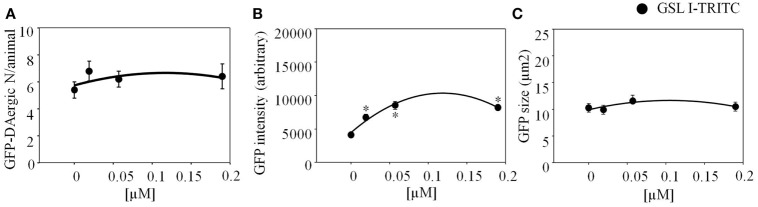
***Griffonia simplicifolia* I (GSL-I)-rhodamine appeared to affect the GFP-DAergic neurons**. **(A)** GSL-I did not affect the number of the GFP DAergic neuron. **(B)** GSL-I increased fluorescence intensity of the neuron at all doses (*P* < 0.05). **(C)** GSL-I did not affect neuron size. *indicates statistical significance.

*Griffonia Simplicifolia* I (GSL-I)-rhodamine (Vector) did not show fluorescence co-localization, and did not affect the number or size of GFP-DAergic-N (Figures 3A,C). The fluorescence intensity of the neuron was increased at all doses (Figure 3B).

**Figure 4:**
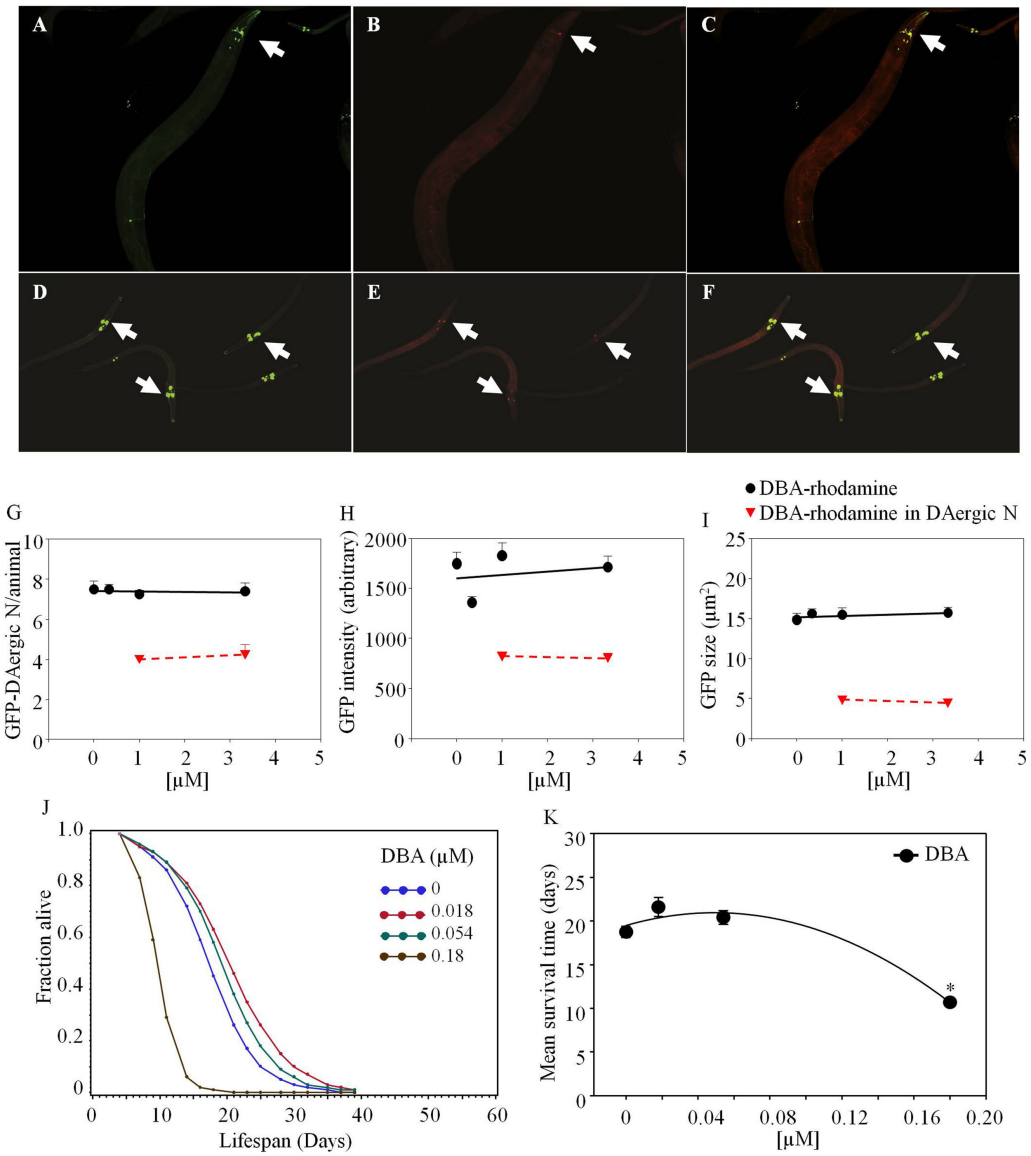
***Dolichos biflorus* agglutinin (DBA)-rhodamine in *C. elegans* co-localized with GFP-DAergic neurons of aged and young nematodes (L3)**. GFP-DAergic neurons in aged animals **(A)** or young animals **(D)**. DBA-rhodamine in GFP-DAergic neurons in aged animals (1.0 × 10^−3^ or 3.33 × 10^−3^ mM) **(B)** or young animals **(E)**. Merged **(A,B)** showing co-localization of the DBA-rhodamine with GFP-DAergic neurons in aged animals **(C)** or young animals **(F)**. **(G)** The number of DAergic neurons was not altered (7.5, *n* = 5, *P* > 0.2) and 50% of them were co-localized with DBA-rb (1.0 × 10^−3^ or 3.33 × 10^−3^ mM, *n* = 3) in the aged animals. **(H)** The fluorescence intensity per GFP-DAergic neuron. **(I)** The size of the GFP-DAergic neurons was not changed. These observations were similarly seen in young animals. **(J)** The APS was increased at lower doses of DBA, and decreased at higher doses. **(K)** Mean survival time of each group. *indicates statistical significance.

**Figure 5:**
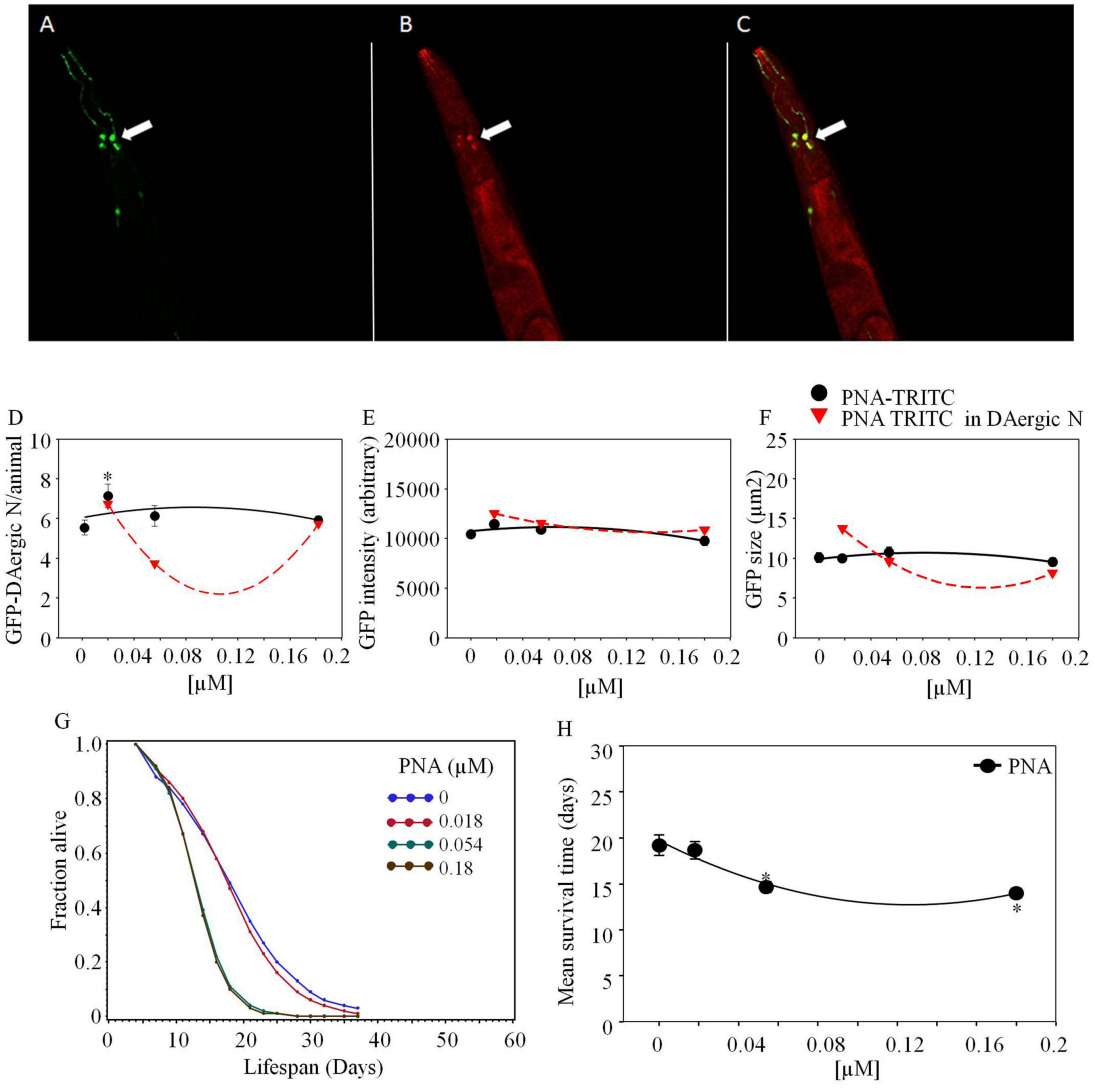
***Arachis hypogaea* agglutinin (PNA)-TRITC post-feeding *C. elegans* co-localized with GFP-DAergic neurons (Leica, TCS SP5, Germany)**. **(A)** GFP-DAergic neurons (green), **(B)** PNA-TRITC in the neuron (red), **(C)** Co-localization of the GFP-DAergic neurons in merged **(A,B)** (yellow). **(D)** Number of GFP-DAergic neurons was increased at low dose (*P* < 0.05). **(E)** The intensity of GFP-DAergic neurons was not altered. **(F)** The size of GFP-DAergic neurons was not altered. **(G)** APS was dose-dependently reduced at all doses. **(H)** Mean survival time of each group. *indicates statistical significance.

*Dolichos biflorus* agglutinin-rhodamine co-localized with GFP-DAergic-N within 3 days after feeding. In aged nematodes, 50% of neurons co-localized with DBA-rhodamine at high doses (Figure 4). The number (Figure 4G), fluorescence intensity (Figure 4H), or size (Figure 4I) of DAergic-N were not altered (*P* > 0.05). In 2-day-old animals, the number of GFP-DAergic-N was similar to aged animals and 65–80% co-localized with DBA-rhodamine (Figures 4E,G). The size of GFP-DAergic-N was reduced in a dose-dependent trend at higher doses of DBA-rhodamine (*P* > 0.05) (data not shown). The fluorescence intensity and size of GFP-DAergic-N in 2-day-old animals were similar to aged animals (Figure 4E,F). The APS was increased at lower doses of DBA, and decreased at higher doses (Figure 4J). Mean lifespan was reduced at the highest dose (1.8 × 10^−4^ mM) from 19 to 11 days (−43%, *P* < 0.05, Figure 4K). Although some experiments were done adding lectin-specific inhibitory sugars to the medium for mitigation of the lectin effects, very high concentrations, up to 200 mM were needed to show any effects, and there were concerns about pleiotropic effects of these sugars fed at high amounts. For example, the animals died in presence of GalNAc (50 mM) within 2 days, possibly because of uridine diphosphate (UDP) depletion (68). Another example of problems with dosing live animals with dietary sugars to offset the lectin effects was that the mean lifespan was reduced by galactose (200 mM) from 19 to 12 days (−36%, *P* < 0.05, Figure 5H). Use of high concentrations of sugars on whole animals had their own effects. Therefore, we have not included data herein using lectin-inhibitory sugars.

*Arachis hypogaea* agglutinin (PNA)-TRITC co-localized with GFP-GAergic neurons after 1-week treatment (1.8 × 10^−5^, 5.4 × 10^−5^, 1.8 × 10^−4^ mM, Figure 5). The size and intensity of GFP-GAergic neurons was not altered. The number of GFP-GAergic neurons was increased at low dose (1.8 × 10^−5^ mM, *P* < 0.05) and reduced at higher doses. APS was dose-dependently reduced at all doses (Figure 5G). Mean lifespan was reduced by higher doses (5.4 × 10^−5^ mM and 1.8 × 10^−4^ mM) from 19 to 15 and 14 days (−24 and −27%, *P* < 0.05, Figure 5H), with an increase at the highest dose (1.8 × 10^−4^ mM) from 12 to 16 days (33%, *P* < 0.05).

### Lectins Altered the GFP-DAergic Neurons Without Co-Localization

Lectins that alter number, GFP-intensity, or size of neurons without observed co-localization are given in Table 2.

**Table 2:** **Lectins alter number, GFP-intensity, or size of DAergic neurons without observed co-localization**.

Fluorescence lectins	Dose (mM)	GFP #		GFP intensity		GFP size	
*Griffonia simplicifolia* I (GSL-I)-rhodamine	5.2 × 10^−4^	↔	*P* > 0.05	↑	*P* < 0.05	↔	*P* > 0.05
	1.7 × 10^−3^						
	5.2 × 10^−3^						

*Pisum sativum* (PSA)-rhodamine (agar)	8.7 × 10^−4^	↔	*P* > 0.05	↓	*P* < 0.001	↓	*P* > 0.05
	8.7 × 10^−3^						

*Pisum sativum* (PSA)-rhodamine (liquid)	4.3 × 10^−5^	↔	*P* > 0.05	↔	*P* > 0.05	↓	*P* > 0.05
	1.3 × 10^−4^			↔	*P* > 0.05	↔	*P* > 0.05
	4.3 × 10^−4^			↓	*P* > 0.05	↔	*P* > 0.05

*Concanavalin A* (Con A)-TRITC (agar)	3.8 × 10^−4^	↔	*P* > 0.05	↔	*P* > 0.05	↓	*P* > 0.05
	1.2 × 10^−3^			↑	*P* < 0.05		
	3.8 × 10^−3^						

*Concanavalin A* (Con A)-TRITC (liquid)	1.9 × 10^−5^	↔	*P* > 0.05	↔	*P* > 0.05	↔	*P* > 0.05
	5.7 × 10^−5^			↔	*P* > 0.05	↓	*P* > 0.05
	1.9 × 10^−4^			↑	*P* < 0.05	↔	*P* > 0.05

*Triticum vulgaris* (WGA)-TRITC	4.6 × 10^−5^	↔	*P* > 0.05	↔	*P* > 0.05	↓	*P* < 0.05
	1.4 × 10^−4^			↔	*P* > 0.05		
	4.6 × 10^−4^			↑	*P* < 0.05		

*Triticum vulgaris* (Succinylated) S-WGA-TRITC (agar)	1.1 × 10^−3^	↔	*P* > 0.05	↓	*P* < 0.05	↔	*P* > 0.05
	1.1 × 10^−2^						

*Triticum vulgaris* (Succinylated) S-WGA-TRITC (liquid)	4.6 × 10^−5^	↓	*P* < 0.05	↑	*P* < 0.05	↓	*P* < 0.05
	1.4 × 10^−4^	↓	*P* < 0.05				
	4.6 × 10^−4^	↔	*P* > 0.05				

*↓ Decreasing trend*.

*↑ Increasing trend*.

*↔ No significant alternation*.

**Figure 6:**
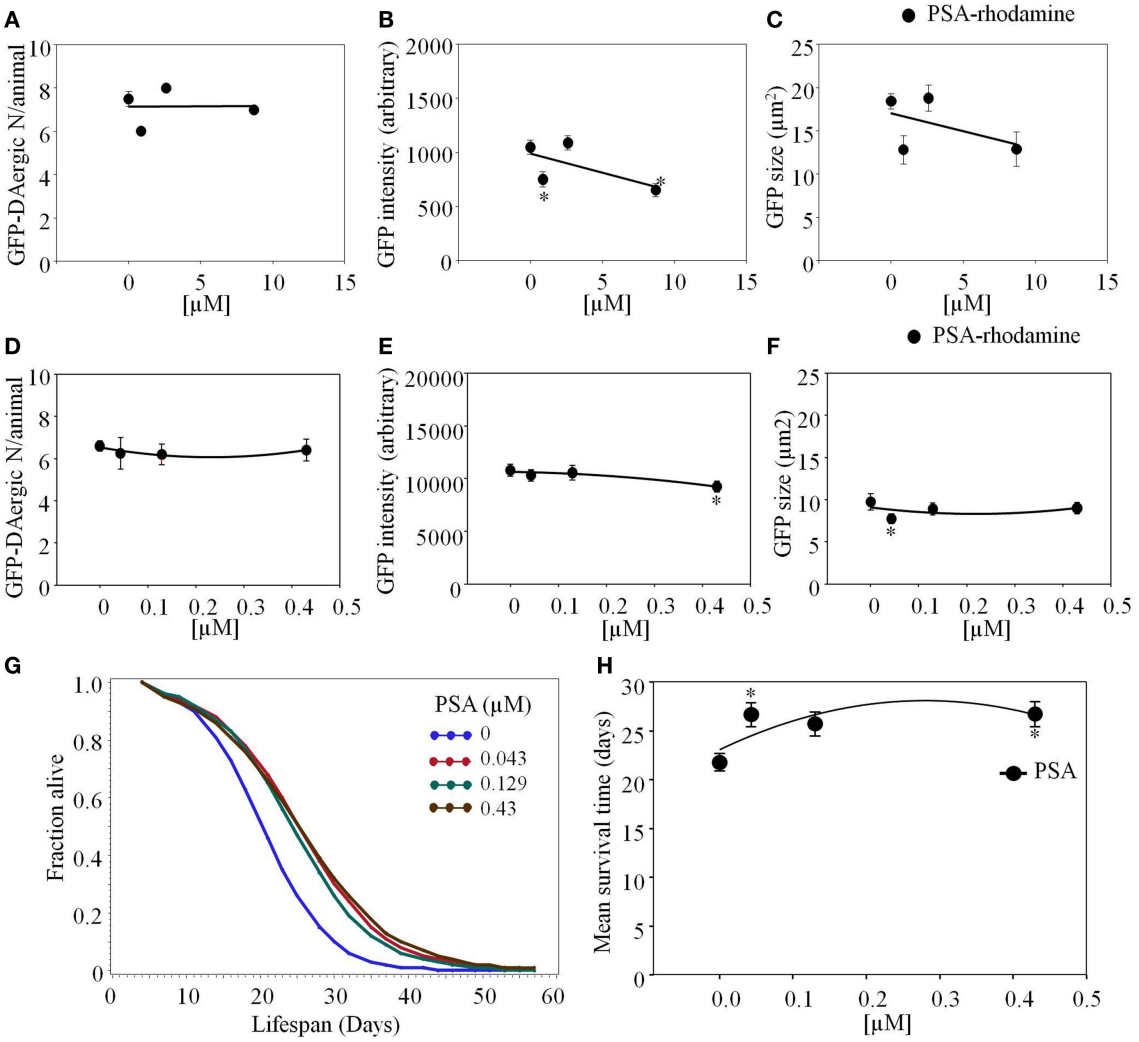
***Pisum sativum* (PSA)-rhodamine affected the GFP-DAergic neurons in *C. elegans***. **(A)** The number of GFP-DAergic neurons per animal was not reduced overall (8.70 × 10^−4^–8.7 × 10^−3^ mM, *n* = 6, *P* > 0.05). **(B)** The fluorescence intensity of GFP-DAergic neurons was decreased at high dose (651 ± 61, 8.7 × 10^−3^ mM). **(C)** The size of GFP-DAergic neurons appeared to be reduced at high dose from 18.4 ± 0.9 μm^2^ (control) to 12.9 μm^2^ (8.70 × 10^−4^ mM, *n* = 3, *P* > 0.05) in a dose-dependent trend. Direct co-localization of PSA-rhodamine with GFP-DAergic neurons was not detected. **(D)** PSA alone did not affect the number of the neurons. **(E)** PSA decreased the intensity of DAergic neurons at the highest dose (*P* < 0.05). **(F)** PSA decreased the size of the DAergic neurons at lowest dose (*P* < 0.05), which was mitigated at the higher dose (*P* > 0.05). **(G)** The APS was increased at all doses. **(H)** Mean survival time of each group. *indicates statistical significance.

*Pisum sativum*-rhodamine feeding did not reduce the number of GFP-DAergic-N (*P* > 0.05, Figure 6A). However, the fluorescence intensity (*P* < 0.001) and size (*P* > 0.05) of GFP-DAT in DAergic-N were dose-dependently decreased with PSA feeding (*P* = 0.8, Figures 6B,C). In liquid culture, the number of the GFP-DAergic-N was not altered (Figure 6D), the intensity was diminished at high dose (4.3 × 10^−4^ mM, *P* < 0.05, Figure 6E), and the size was reduced at low dose (4.3 × 10^−5^ mM, *P* < 0.05, Figure 6F). The APS was increased at all doses (Figure 6G). Mean lifespan was increased by lower doses (4.3 × 10^−5^ mM and 4.3 × 10^−4^ mM) from 22 to 27 days (22 and 23%, *P* < 0.05, Figure 6H).

**Figure 7:**
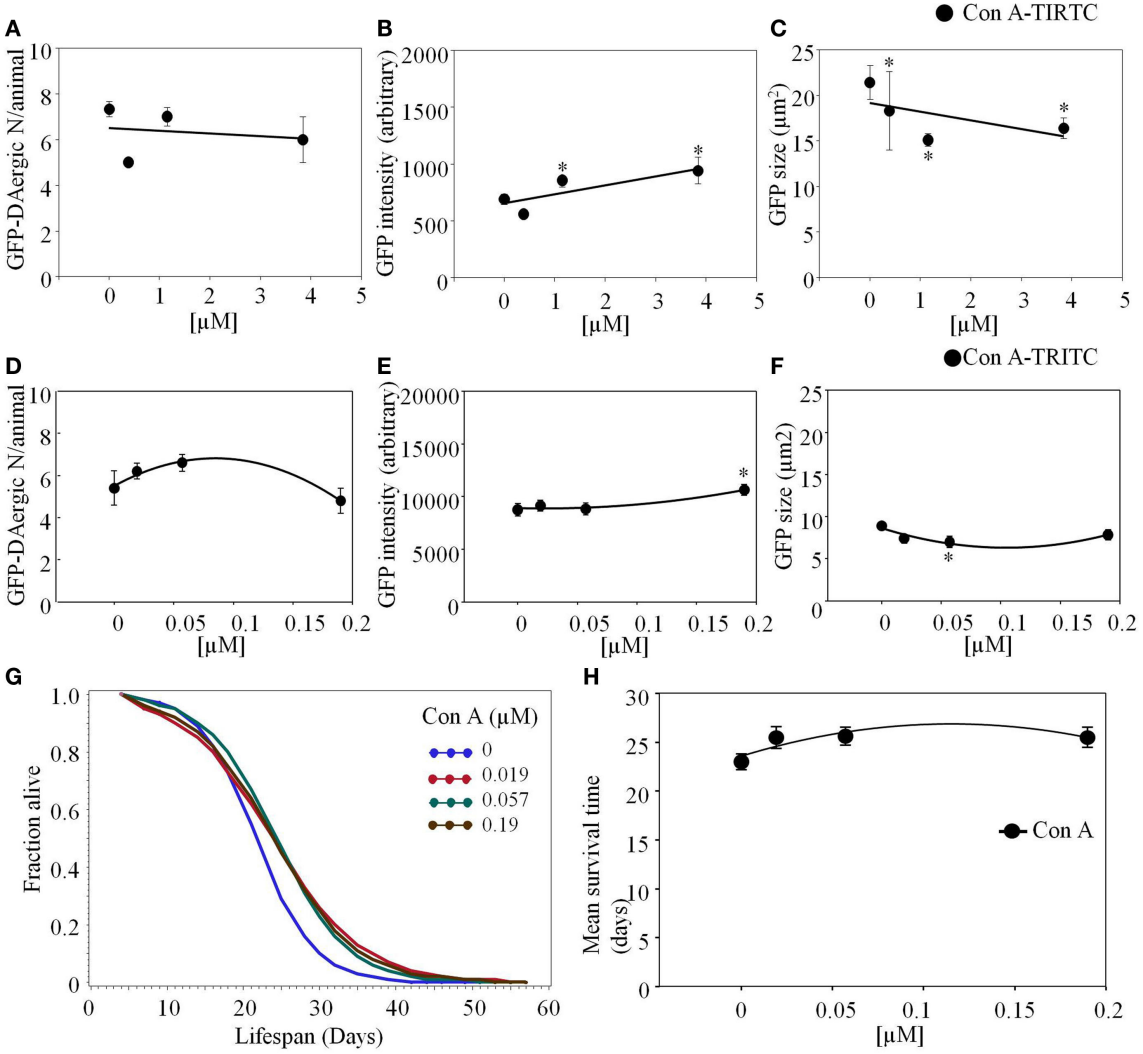
***Concanavalin A* (Con A)-TRITC in *C. elegans* appeared to have a mild effect on the GFP-DAergic neurons**. **(A)** The number of GFP-DAergic neurons per animal was slightly reduced at the high dose (7 ± 0.4, *P* > 0.05). **(B)** The fluorescence intensity per GFP-DAergic neuron was dose-dependently increased from 691 ± 45 (control, *n* = 3) to 942 ± 118 (3.84 × 10^−3^ mM, *n* = 4, *P* < 0.05). **(C)** The size of GFP-DAergic neurons was significantly reduced from 21.4 ± 1.8 μm^2^ (control) to 15.1 ± 0.7 μm^2^ (1.15 × 10^−3^ mM, *n* = 4, *P* < 0.05). Direct co-localization of Con A-TRITC with GFP-DAergic neurons was not detected. In liquid culture, Con A-TRITC in *C. elegans* appeared to have a mild effect on the GFP-DAergic neurons. **(D)** The number of the GFP-DAergic neuron was not affected (*P* > 0.05). **(E)** The fluorescent intensity of the DAergic neuron was increased at the highest dose (*P* < 0.05). **(F)** The area of GFP-DAergic neurons was decreased at middle dose (*P* < 0.05). **(G)** The APS was increased at all doses. **(H)** Mean survival time of each group. *indicates statistical significance.

*Concanavalin A* (Con A)-TRITC appeared to have a mild effect on the GFP-DAergic-N reducing the number of GFP-DAergic-N in a dose-dependent trend (Figure 7A). The fluorescence intensity of GFP-DAT protein image in DAergic-N was dose-dependently increased by Con A feeding (*P* < 0.05, Figure 7B). The apparent size of GFP-DAergic-N was significantly reduced (*P* < 0.05, Figure 7C). Con A-TRITC did not alter the number of GFP-DAergic-N in liquid culture (*P* > 0.05, Figure 7D), increased the intensity at high dose (1.9 × 10^−4^ mM, *P* < 0.05, Figure 7E), and decreased the size of neuron at middle dose (5.7 × 10^−5^ mM, *P* < 0.05, Figure 7F). The APS was increased at all doses (Figure 7G). Con A did not affect the mean lifespan (Figure 7H).

**Figure 8:**
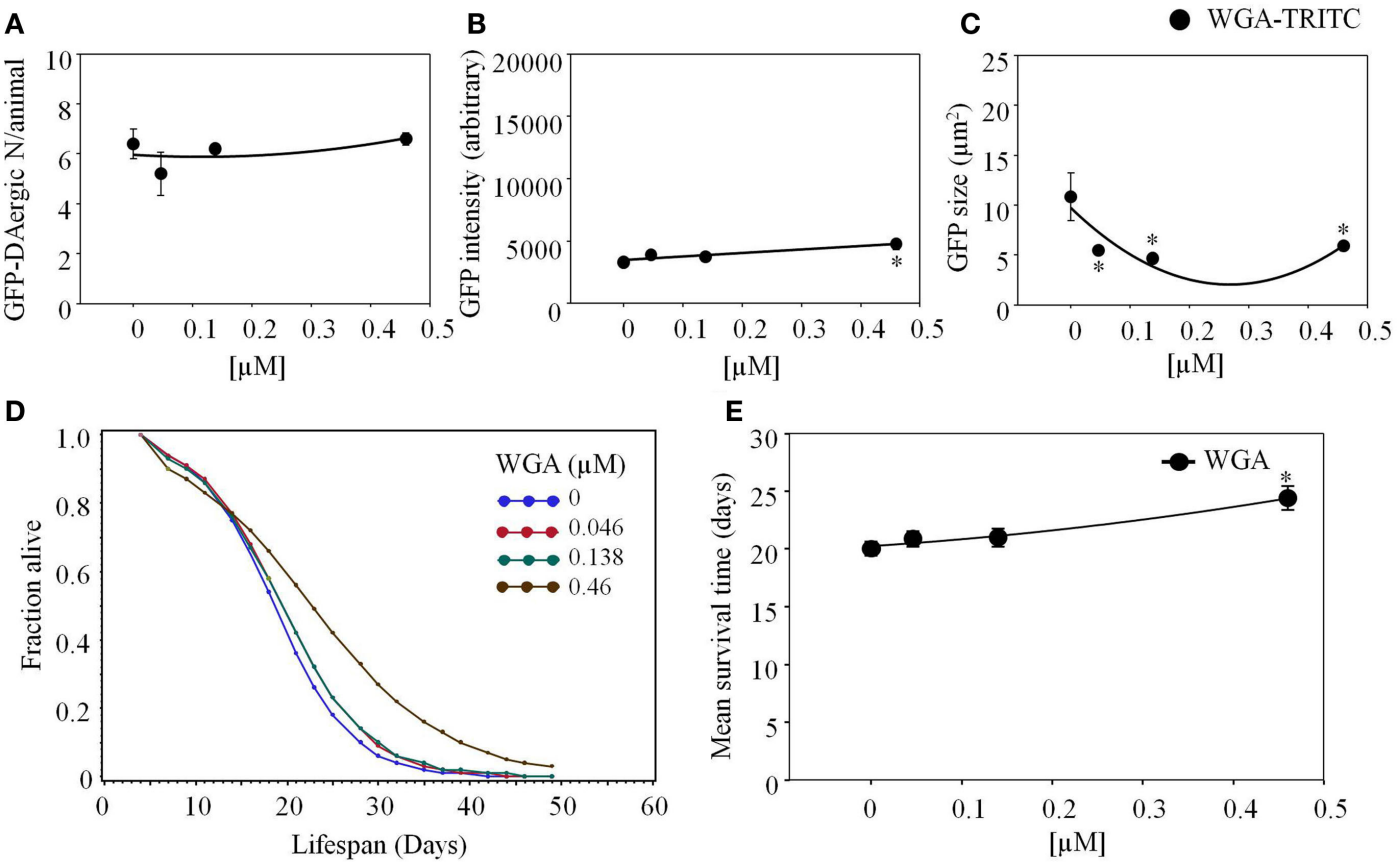
***Triticum vulgaris* (WGA)-rhodamine affected the intensity and area of DAergic neurons in liquid culture**. **(A)** WGA did not affect the number of DAergic neurons **(B)** WGA increased the intensity of the GFP-DAergic neurons. **(C)** The area of the DAergic neurons was reduced at all doses (*P* < 0.05). **(D)** The APS was increased at all doses. **(E)** Mean survival time of each group. *indicates statistical significance.

*Triticum vulgaris* (WGA)-rhodamine or WGA-TRITC was not detected as transported (Figure 8). The intensity of DAergic-N was increased (*P* < 0.05, Figure 8A), while the size of the neurons was decreased (*P* < 0.05) at the highest dose (4.6 × 10^−4^ mM, Figure 8B). The APS was increased at all doses (Figure 8D). Mean lifespan was increased at the highest dose (4.6 × 10^−4^ mM) from 20 to 24 days (22%, *P* < 0.05, Figure 8E).

**Figure 9:**
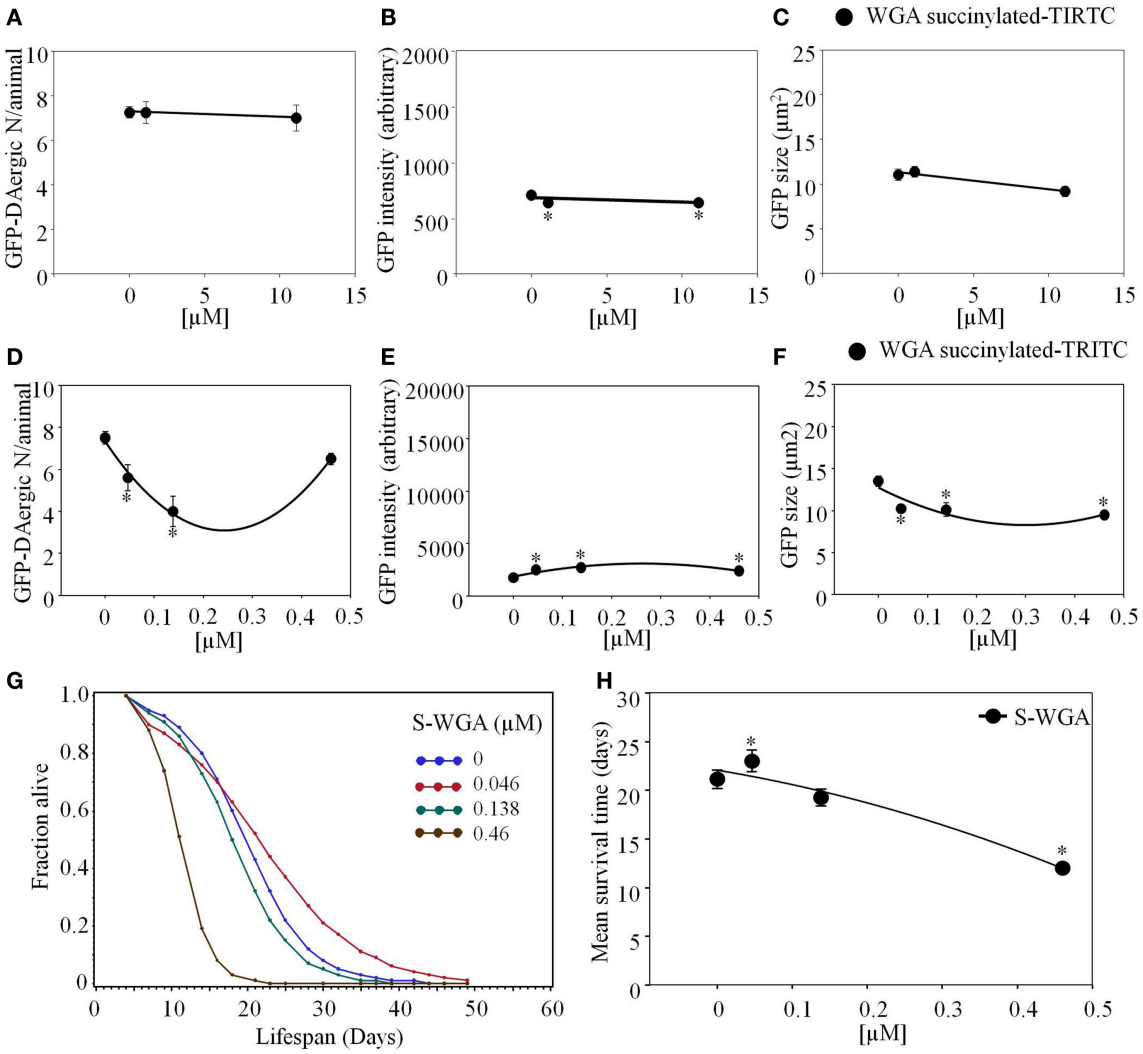
***Triticum vulgaris* (Succinylated) S-WGA-TRITC affected *C. elegans* GFP-DAergic neurons**. **(A)** The number of GFP-DAergic neurons per animal was similar to the effect of WGA-TRITC (7.3–7.0, *n* = 4, *P* > 0.5). **(B)** The fluorescence intensity was reduced from 735 ± 26 (control) to 667 ± 25 (1.11 × 10^−2^ and 11.12 × 10^−3^, *n* = 4, *P* < 0.05). **(C)** The size of GFP-DAergic neurons were slightly reduced at the high dose only to 9.2 ± 0.6 μm^2^ (11.12 × 10^−2^, *n* = 4, *P* = 0.4). Direct co-localization of WGA-TRITC with GFP-DAergic neurons was not detected. S-WGA-TRITC affected the GFP-DAergic neurons in liquid culture. **(D)** The number of the GFP-DAergic neurons was decrease at low doses. **(E)** The fluorescence intensity of the GFP-DAergic neurons was increased. **(F)** The size of the GFP-DAergic neurons was decreased. **(G)** The APS was increased at a low dose, and decreased does-dependently at higher doses. **(H)** Mean survival time of each group. * indicates statistical significance.

*Triticum vulgaris* (Succinylated) S-WGA-TRITC feeding had a similar effect as WGA-rhodamine. The number of DAergic-N did not show a significant change (Figure 9A), however, fluorescent intensity of the GFP-DAergic-N was decreased (*P* < 0.05, Figure 9B). The size of GFP-DAergic-N was slightly reduced in a dose-dependent trend (*P* = 0.4, Figure 9C). In these cases, it is not clear whether the lectin has some undefined indirect effect or that there is a direct effect by undetectable amounts of lectin. S-WGA-TRITC feeding *C. elegans* did not show co-localization in liquid culture. The number of the GFP-DAergic-N was decreased at lower doses (4.6 × 10^−5^ mM and 1.4 × 10^−4^ mM, *P* < 0.05, Figure 9D). The fluorescent intensity of GFP-DAergic-N was increased at all doses (*P* < 0.05, Figure 9E). The size of GFP-DAergic-N was decreased at all doses (*P* < 0.05, Figure 9F). The APS was increased at a low dose, and decreased does-dependently at higher doses (Figure 9G). The mean lifespan was increased at a low dose (4.6 × 10^−5^ mM) from 21 to 23 days (9%, *P* < 0.05), and decreased at the highest dose (4.6 × 10^−4^ mM) to 12 days (−43%, *P* < 0.05, Figure 9H).

## Discussion

Nine examined plant dietary lectins were conjugated to TRITC or rhodamine. Lectins were tested in the *in vivo C. elegans* (*egIs1*[*Pdat-1:GFP*]) model. An elevated GFP-DAT that is expressed under control of the DAT gene promoter shows enhanced DAT expression and trafficking by the promoter, transcription factor, and nuclear receptor (69).

Carbohydrate-binding protein toxins are known to survive and traverse the gut intact, as an acutely toxic substance and can induce serious life-threatening illness in humans and animals. Distance pathogenicity of botulinum toxin, as well as cholera toxin, impairs the CNS (70). The present study was aimed at a new, surprising property of lectins based upon the hypothesis that lectins may be transported directly by gut absorption to local neurons and transported axonally to distal neurons where they have an anatomical and potentially a physiological pathophysiological effect. Fluorescent intensity and co-localization of lectins was observed to suggest transport to GFP-DAergic-N. Number and area changes of GFP-dopamine receptor fluorescence in DAergic-N were observed to be an effect of the lectins. For the fact that multiple system atrophy features the accumulation of α-SYN in glial cytoplasmic inclusions (71–73), involvement of other pathways such as indirect effects by interaction of lectins with glial cells that affected DAergic-N may be possible. Lectins have been used for histochemistry and neuronal tracing only, but were not previously associated with neuronal toxicity (26, 74, 75).

### The Occurrence and Intensity of Individual Fluorescently Labeled Lectins in GFP-DAergic-N Detected by Co-Localization

Four lectins (PHA-E, BS-I, PNA, or DBA) appeared to co-localize with a subgroup of GFP-DAergic-N, while some other lectins had effects where co-localization was not observed. Because some effects were seen in neurons where lectins were not detected, this may be due to undetectable amount of transported lectin, or some unexplained indirect effect. Thus, the number and GFP-DAT image of neurons were also evaluated, even when the fed lectin was not detected in the neurons. The lack of observation of fluorescence in the neurons using other fed lectins (which, however, seemed to affect these neurons) may be due to a variety of characteristics. For instance, a critical window for the lectin to be detectable may have been missed, the lectin may have been partially degraded, losing the fluorophore, but still retaining neuron-effective activity, or, more likely, undetectable levels of the lectin have observable activities. Future studies with ELISA, other immunocytochemical studies or radiolabeling may confirm the transport of small amounts of these specific lectins where an effect is observed without fluorescence co-localization.

#### Lectin-Caused Differences in the Number of GFP-DAergic Neurons

Four lectins [GSL-I (BSL I), PHA-E, Con A, and PSA] reduced the number of DAergic-N, with GSL-I having the greatest effect. Toxicity of some lectins and newly discovered side effects of ingestion of PHA and WGA lectins in human and animals have been observed, mitigated by sucrose feeding (76–78). In the present study, PHA-E-rhodamine appeared to be transported intracellularly, probably by axonal transport after ingestion, to *C. elegans* GFP-DAergic-N causing a reduction in their number. This reduction was inversely proportional to the size of the observed image of PHA-E-rhodamine fluorescence co-localized with the neurons. Thus, the greatest area of PHA-E-rhodamine co-stains with the lowest observed number of GFP-DAergic-N. In addition, both fluorescence intensity and size of the GFP-DAergic-N were significantly reduced, suggesting a possible toxic effect of cytoplasmic PHA-E. This observation is in agreement with other studies showing that PHA can damage intestinal epithelial cells (32, 78), which was prevented or reversed by a PHA-E inhibitor sucrose (78). Interestingly, PHA-E did not show a significant decrease in the number and size of GFP-neurons in *C. elegans* but demonstrated decreased expression of GFP-DAT fluorescence intensity. PSA, in some studies, has been shown to be essentially non-toxic in mice both *in vivo* and *in vitro* (79). However, short-term toxicity measurements in these studies do not include more subtle, possible long-term effects of neuronal damage.

### Lectin Feeding Effects on the Fluorescent Intensity of GFP-DAergic Neurons

Three lectins (PHA-E, PSA, or S-WGA) reduced GFP-DAT fluorescence in DAergic-N suggesting damage to DAT, while BS-I, GSL-I, or Con A induced an increase indicating a promotion of DAT in the DAergic-N. WGA slightly reduced the fluorescent intensity of GFP-DAT in DAergic-N, while DBA was inactive. Although WGA did not affect the number of GFP-DAergic-N in the present study, and in other laboratories, in the *in vivo* rat gut lumen, reduced expression of heat shock proteins resulting in lowered protection and greater permeability of epithelial cells. WGA also increases thrombin in human platelets, and escalates adipogenesis in mesenchymal cells of the mouse limb bud *in vitro* by unknown mechanisms (25, 77, 80, 81).

In liquid culture, individual lectins affect the number and intensity of GFP-DAergic-N in a different manner. GSL-I, Con A, WGA, and PSA mildly affected the number of GFP-DAergic-N. The intensity of GFP-DAergic-N was increased by GSL-I, Con A, and WGA, while decreased by PSA. The number of GFP-DAergic-N was reduced, while the intensity of GFP-DAergic-N was increased by S-WGA in lower doses. PNA did not affect the intensity of the GFP-DAergic-N, while increased the number of neurons at lower doses.

### Sizes of GFP-DAergic Neurons

PHA-E or Con A significantly diminished the size of GFP-DAergic-N, while BS-I, PSA, or S-WGA slightly reduced the size. Whether these effects signify damage to the neurons is not known. Increased neuron size of a subgroup of GFP-DAergic-N, however, was also observed with DBA or WGA, which may have promoted DAT expression, however, whether decrease or increase in the apparent size of neurons has a physiological effect, or indicates that lectin-mediated damage is not yet known.

The effect of lectins on the number of GFP-DAergic-N appeared in the following order PHA-E > GSL-I > BS-I > Con A > PSA > S-WGA. An elevation effect of lectins seemed to be in the order of DBA > BS-I > WGA > GSL-I, and ConA. It is now well known that O-linked β-*N*-acetylglucosamine (*O*-GlcNAc) is a common epitope on cytoplasmic and some nuclear proteins sharing common features with protein phosphorylation [see review (82)]. Although very difficult to detect due to substoichiometric amounts, *O*-GlcNAc occurs exclusively within the nuclear and cytoplasmic compartments of the cell and responds to external signaling, such as mitogen or antigen activation, by altering Ser- and Thr-phosphoprotein profiles (83–84). The protein modification is modulated by *O*-GlcNAcase and *O-*GlcNAc transferase, and even glucose levels modulate the *O*-GlcNAc cycling rate. Characteristics of half-life in *O*-GlcNAc cycling (≤1 min) have been linked closely to diabetes, cardiovascular disease, neurodegenerative disorders, and cancer. Many protein O-GlcNAc modifications occur in nucleocytoplasmic compartments across plant and animal species, including humans [see review (82)]. WGA and other GlcNAc binding lectins, if present in the cytoplasm, may affect this balance (our hypothesis). PNA strengthens extracellular matrixes by promoting production of proteoglycan in mouse chondrocytes *in vitro* (81). Similar to PNA, Con A has also been reported to strengthen extracellular matrixes in mouse chondrocytes *in vitro* (81). In our study, Con A altered GFP-DAergic-N by reducing the area of GFP-DAT fluorescence.

Some specific beneficial activities of a variety of lectins have been reported (81, 85). In our studies, DBA was observed in the GFP-DAergic-N that had the effect of increasing the observed area of the GFP-labeled DA transporter. This increase may suggest enhanced DAT expression and trafficking, where GFP is expressed under the DAT promoter (69, 86). A major adverse effect of DBA lectin has not been reported elsewhere. In fact, DBA significantly facilitates cartilagenesis and osteogenesis in mouse limb bud mesenchymal cells *in vitro* (81).

BS-I and GSL-I displayed similar activities. Significantly, BSI I dose-dependently reduced the intensity of GFP-DAergic-N. Functionally, BS-I containing 5-Hydroxytryptophan (5-HTP), a catecholamine neurotransmitter, only binds to a subgroup of small dorsal root ganglion neurons, which are of the C-nociceptor type (C-fiber nociceptive or unresponsive) neurons. These C-nociceptor neurons mediate visceral pain and express receptors to BS-I isolectin-B4 (IB4) (87–89). BS-I may increase thrombin in human platelets (80). One possible mechanism that might be related is over-excitation of neurons caused by excessive glutamate neurotransmission being neurotoxic, which may cause neuronal death (90). Indeed, glutamatergic stimulation may indirectly induce DAergic neuronal death *via* unbalanced calcium homeostasis and oxidative stress, *in vitro* or *in vivo* (91). Some of these systems could be altered by either sugar-binding or other unexplored properties of lectins.

In liquid culture, PSA, Con A, WGA and S-WGA decreased the area of GFP-DAergic-N. GSL-I and PNA did not show significant change.

### Elevated Fluorescent Intensity and Size of DAergic Neurons

These alterations may reflect a relationship with the insulin receptor and DAT. Glucose provides a vital energy source for brain and clearly modulates neuronal function (92, 93). In *C. elegans*, hyperglycemia reduces APS, related to human diabetes. These relationships in our study, however, may represent some signaling interaction of glycemia/insulinemia and DAT. As with other catecholamine neurotransmitters, inhibitory neurotransmitters are inversely proportional to glycemia, and DA kinetics is sensitive to hypoglycemia in a complex manner (94). In rodents, insulin receptors and DAT are densely present in *substantia nigra*, insulin may increase DAT mRNA expression, and glycemic index is inversely associated with the risk of PD (95, 96).

### Lectins Affect Average Probability of Survival of *C. elegans*

Lectins can be categorized into three groups based on their effects on APS of *C. elegans*. (1) PHA-E, DBA, and S-WGA showed J-shaped effects on APS that was increased with lower doses, while decreased at higher doses. (2) PSA, Con A, and WGA demonstrated augmented effects on APS that was increased at all doses in a dose-dependent manner. (3) GSL-I and PNA decreased the APS dose-dependently.

Neurotoxins such as botulinum toxin or ricin bind to specific receptors on cell membrane, to be internalized, and exert toxic effects (97, 98). Similarly, lectins bind to carbohydrate ligands of targeted cells to be functional, producing effects. Specific sugars may competitively bind and inhibit lectins uptake (99). Structurally similar lectins might share similar binding receptors and compete for each other’s binding sites. The complex GI environment, including nutrients and cellular environment, might alter the absorption of certain lectins and their potential biological consequences (100).

In a recent Danish report, patients who had vagal nerves removed 20 years earlier had 40% lower incidence of PD than control populations. If dietary proteins are one potential etiology for PD, by transport to neurons from the gut, as hypothesized here, removal of the vagal nerve would have prevented or reduced this etiology pathway. Symptoms of motor impairment are typical in PD patients, and dysfunction of aspects of the autonomic nervous system are often underrated, such as GI motility (101), rapid eye movement (102), and so on. The current study indicates potential transport of some dietary plant lectins from the GI tract to the DAergic-N in *C. elegans*, with direct or indirect effects on these neurons and diverse effects on APS. This observation may be related to the Braak and Hawkes’ hypothesized unknown etiologic agent for PD or related, for example, to damaged DAergic-N that have been found in PD (40, 47). If related, the process may be gradual, may be additive, related to the frequency of consumption of certain lectins, and may be determined by the association of lectins with other factors. Certainly, there is potential for inputs from individual genetic susceptibility, varying sugar structures profiles in different cell membranes, the receptivity to endocytosis, a disorder or leakage of the GI lining, and dietary content. Our observations are a tantalizing possible explanation for why dietary plants have been linked to a risk of developing PD.

## Author Contributions

JZ designed the study; RL and JK gave advice regarding the design and conduct of the study; JZ, MW, WW, BA, and MK conducted data acquisition and data analyses; JZ, MW, WW, JK, and RL participated in drafting the manuscript; all of the authors contributed to review and revision of the submission.

## Conflict of Interest Statement

The authors declare that the research was conducted in the absence of any commercial or financial relationships that could be construed as a potential conflict of interest.

## Footnotes

^1^http://www.nobelprize.org/nobel_prizes/medicine/laureates/1908/
